# Clinical and Molecular Characteristics of Foveal Sparing Phenotype in Chinese Patients With Inherited Retinal Diseases

**DOI:** 10.1167/tvst.15.2.18

**Published:** 2026-02-17

**Authors:** Zilin Wang, Tong Li, Jieqiong Chen, Yiqing Sun, Huixun Jia, Junran Sun, Xiaodong Sun

**Affiliations:** 1 Department of Ophthalmology, Shanghai General Hospital, Shanghai Jiao Tong, University School of Medicine, Shanghai, People's Republic of China; 2 Shanghai Gene Therapy Center, Shanghai, People's Republic of China; 3 Shanghai Key Laboratory of Fundus Diseases, Shanghai, People's Republic of China; 4 Eberly College of Science, Penn State University, University Park, State College, PA, USA; 5 Shanghai Engineering Center for Visual Science and Photomedicine, Shanghai, People's Republic of China; 6 National Clinical Research Center for Eye Diseases, Shanghai, People's Republic of China

**Keywords:** inherited retinal diseases (IRDs), foveal sparing, optical coherence tomography (OCT), residual ellipsoid zone (EZ), imaging biomarker

## Abstract

**Purpose:**

The purpose of this study was to evaluate the clinical characteristics of the foveal sparing phenotype among patients with inherited retinal diseases (IRDs) and to identify the predictive imaging markers for visual prognosis.

**Methods:**

Consecutive patients with definitive clinical and genetic diagnoses of IRD who first visited our clinic from November 2021 to December 2022 and had high-quality spectral-domain optical coherence tomography (SD-OCT) images were included in this retrospective cohort. The foveal sparing phenotype was defined by foveal preservation on autofluorescence (FAF) and OCT images. Best-corrected visual acuity (BCVA) and dimensions of residual structures of outer retinal layers on OCT and FAF images were measured and analyzed. Spearman correlation analysis was performed to evaluate the correlation between retinal imaging features and BCVA. Receiver operating characteristic (ROC) curve was performed to assess the predictive performance of residual ellipsoid zone (EZ) area on OCT.

**Results:**

A total of 601 eyes of 308 Chinese patients with IRD were enrolled. Foveal sparing phenotype was observed in 195 (32.4%) eyes of 105 (34.1%) patients. There were 46.7% of cases with *USH2A*-related retinopathy and 76.9% *EYS*-related retinopathy presented foveal sparing phenotype, whereas their structural integrity showed no significant difference. Spearman correlation analyses revealed significant association between residual EZ area (*P* < 0.01) and BCVA. ROC curve analysis demonstrated that the residual EZ area at initial diagnosis could predict the degree of BCVA deterioration within 2 years (area under the curve [AUC] = 0.70).

**Conclusions:**

Our findings indicate that the foveal sparing phenotype is associated with better visual prognosis and is more frequently observed in IRDs associated with *EYS* and *USH2A* mutations. The residual EZ area on OCT can serve as a predictor of the timeframe for central vision deterioration to blindness in patients with IRD.

**Translational Relevance:**

Residual EZ area on OCT can serve as a predictive biomarker to support clinical decision making in monitoring and managing the progression of IRD.

## Introduction

Inherited retinal diseases (IRDs) are a group of monogenic retinal diseases that lead to irreversible photoreceptor degeneration and vision loss with a prevalence of approximately 1 in 2000 to 4000 individuals. Over 300 genes and 50 major subtypes have been identified in studies of more than 2 million patients worldwide.^1^^,^^2^ However, limited therapeutic options for IRDs resulted in severe visual impairment in young patients and legal blindness in working-age adults, presenting substantial economic and societal challenges.^3^^–^^5^ Although Luxturna stood out as the first US Food and Drug Administration (FDA)-approved gene therapy specifically for patients with IRD,^6^ no other gene therapy products for IRD have gained approval to date.^6^^,^^7^ The main challenges were the genotype-phenotype heterogeneity and limited knowledge of the natural history of IRD.^1^^,^^8^^–^^10^ Thus, it is essential to elucidate the genotype-phenotype correlations of IRDs, as well as to investigate the associated markers for visual prognosis.

Foveal sparing is a relatively common phenotype in retinal dystrophies including IRDs and geographic atrophy (GA).^11^^–^^14^ It was characterized by the preserved fovea surrounded by atrophy, and was closely associated with better central vision and late-onset of the disease.^15^^–^^18^ However, the characteristics and prevalence of foveal sparing among IRDs caused by different pathogenic genes have not been investigated. To better illustrate the natural course of IRDs, the width of the residual ellipsoid zone (EZ) band and the intensity of fundus autofluorescence (FAF) have been commonly used as imaging biomarkers to evaluate diseases progression.^19^^–^^22^ Previous studies have also indicated that the residual EZ area is consistently correlated with visual function,^23^^,^^24^ however, its relationship with longitudinal changes in visual acuity in patients with IRD has not been thoroughly explored. Thus, it is necessary to explore whether the foveal sparing area measured on spectral-domain optical coherence tomography (SD-OCT) could serve as a reliable predictor of disease progression and prognosis in IRDs.

In this study, we conducted a retrospective study to analyze structure-genotype relationship of foveal sparing in IRD and established an OCT-based predictor of the timeframe for central vision deterioration to blindness in patients with IRD. These findings are critical for elucidating patterns of disease progression and identifying optimal therapeutic windows.

## Methods

### Patient Selection

This is a retrospective study of Chinese patients with IRD evaluated in Shanghai General Hospital from November 2021 to December 2022. This research was approved by the Institutional Review Board of Shanghai General Hospital and was conducted in accordance with the principles outlined in the Declaration of Helsinki (NCT03691168).

All patients enrolled in the cohort underwent basic ophthalmic examinations, including best-corrected visual acuity (BCVA), FAF, and OCT. Patients with clinical and fundus findings suggestive of IRD underwent genetic testing, including targeted Sanger sequencing, next-generation sequencing, retinal dystrophy gene panel assessments, and whole-exome or whole-genome sequencing as part of routine diagnostics. Segregation analysis was performed in existing family members to confirm the inheritance pattern. The patient selection process is detailed in Supplementary Figure S1. The inclusion criteria were as follows:

1) Identification of the causative genetic variants;
2) A definitive diagnosis established by two experienced ophthalmologists based on clinical characteristics;
3) SD-OCT provided a clear view of the structural features of the retinal layers, sufficient for analysis.

The exclusion criteria were as follows: patients with known non-genetic causes of retinal pathology (e.g., trauma) and patients whose ocular conditions interfered with obtaining reliable ophthalmic imaging or clinical data.

**Figure 1.**
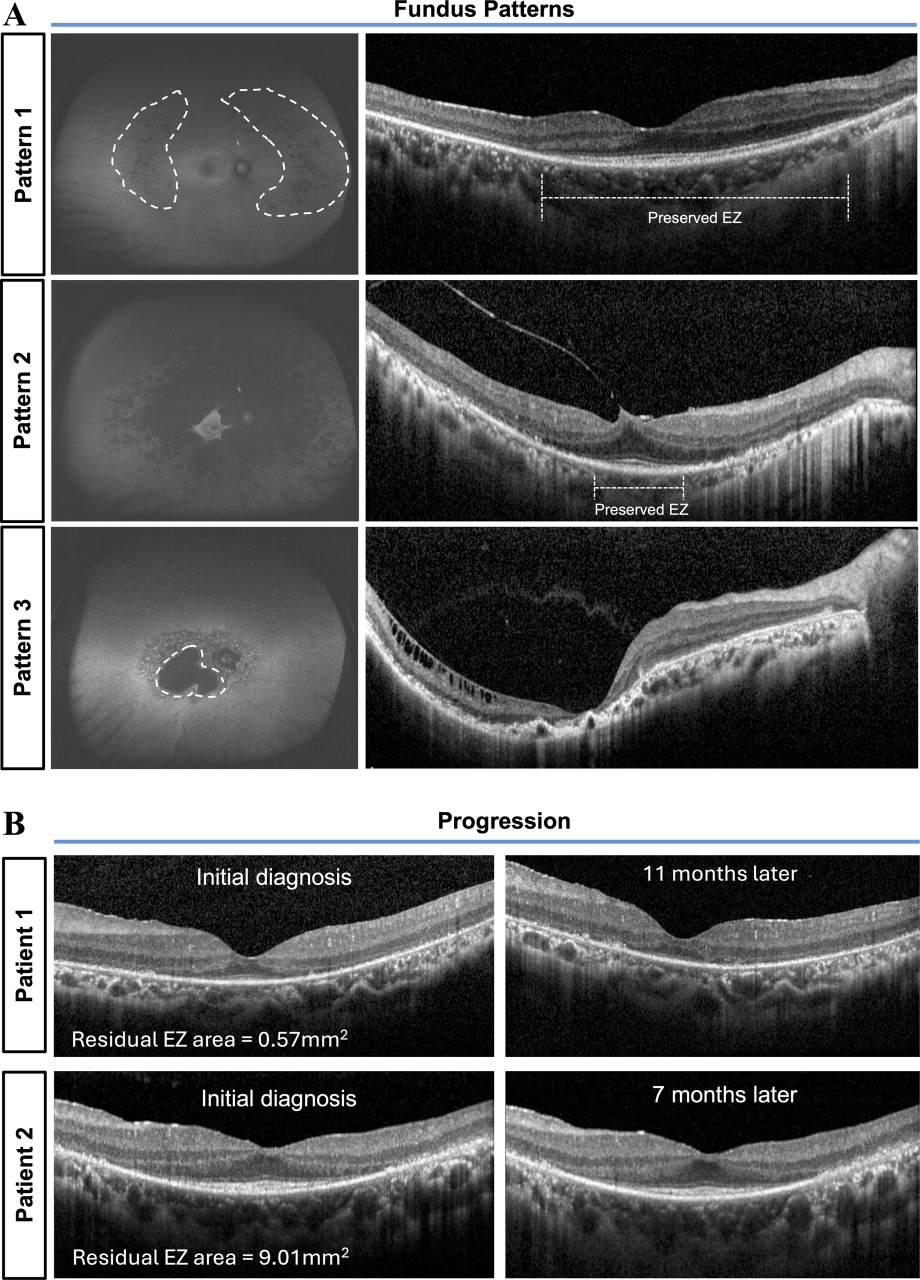
**Foveal sparing patterns and longitudinal changes on OCT images.** (**A**) Classification of foveal sparing patterns on FAF images and the corresponding OCT findings. Pattern 1 = fovea completely spared; pattern 2 = fovea partly spared; and pattern 3 = fovea completely involved. Preserved EZ bands were highlighted on OCT images. (**B**) Longitudinal OCT assessment of foveal sparing in two unrelated patients with *EYS* variants. Patient 1 was a 23-year-old patient with baseline BCVA of 0.52 logMAR; after 11 months, BCVA declined to 0.7 logMAR, exceeding the 0.1 logMAR threshold for significant deterioration. Patient 2 was a 49-year-old patient with baseline BCVA of 0.0 logMAR; after 7 months, BCVA remained stable at 0.05 logMAR. Both patients had a disease duration of 4.5 years at the initial visit. EZ, ellipsoid zone; FAF, fundus autofluorescence; OCT, optical coherence tomography.

In the present study, the foveal region was anatomically defined as a 3-mm diameter circular zone, encompassing both the fovea and parafovea. Foveal sparing was defined as the presence of preserved autofluorescence on FAF imaging and a corresponding preserved EZ band on SD-OCT imaging within the foveal region, as illustrated in Figure 1A. Representative examples are shown in Figure 1B. Choroidal atrophy was defined by choroid thickness less than 125 µm.^25^

Retinitis pigmentosa (RP) phenotype was characterized by arteriolar attenuation, retinal pigmentary changes, and waxy pallor of the optic disc on fundus photography. The causative genetic variants included mutations of *USH2A*, *EYS*, *RP1*, *RPGR*, *PDE6B*, *CNGB1*, etc.^3^ A total of 21 eyes showing foveal sparing from 12 patients who had undergone complete prospective follow-up over 2 years were included for subsequent receiver operating characteristic (ROC) curve analysis. BCVA change more than 0.1 logMAR (1-line step) compared to the initial diagnosis was considered to be significant.^26^^,^^27^

### Clinical Examinations and Image Assessments

Foveal sparing refers to the phenomenon that atrophy lesion surrounds the intact and functioning fovea until later stages of the disease.^11^ The preservation of the foveal outer retinal structure was assessed by external limiting membrane (ELM), EZ, and interdigitation zone (IZ) in the foveal region on a 19-line SD-OCT scan. The residual EZ length was defined on a 19-line scan OCT as the length between the two endpoints where the EZ band reflectivity disappeared (highlighted in Fig. 1A). Specifically, the maximum preserved EZ length was defined as the longest continuous segment of the EZ band observed across all 19-line horizontal OCT scans. The residual EZ area was then calculated using the following formula: residual EZ area = (maximum horizontal EZ length) × (vertical EZ length).

Atrophic lesions were defined as areas exhibiting over 50% darkness on FAF images.^16^^,^^19^^,^^28^ Representative examples illustrating the classification of FAF patterns based on foveal characteristics have been provided in Figure 1A. Using the patterns of hypo-fluorescence observed in FAF, we categorized the fundus conditions into three distinct types (see Fig. 1A)^15^^,^^29^:

1. Pattern 1: Fovea completely spared;
2. Pattern 2: Fovea partly spared;
3. Pattern 3: Fovea completely involved.

The field of view was set to over 200 degrees with a minimum resolution of 768 × 768 pixels and centered on the fovea of FAF. Measurement of atrophy area was performed using the QuPath-0.4.3 (http://qupath.github.io)^30^ and calculated as previously described.^31^

### Statistical Analysis

All data were analyzed by SPSS software version 23 (IBM SPSS Statistics, Chicago, IL). The main outcomes are presented as mean ± standard deviations. Samples from different categories were compared by Student's *t*-tests and ANOVA. Chi-squared (*n* > 5) and Fisher's exact tests (*n* ≤ 5) were used to compare frequency and distribution differences. Spearman correlation analysis was used to identify associations between BCVA and fundus imaging features. ROC curve analysis was used to evaluate the predictive performance of various clinical features, and determine optimal threshold values for classification. Results with *P* values < 0.05 were regarded as statistically significant.

## Results

**Figure 2.**
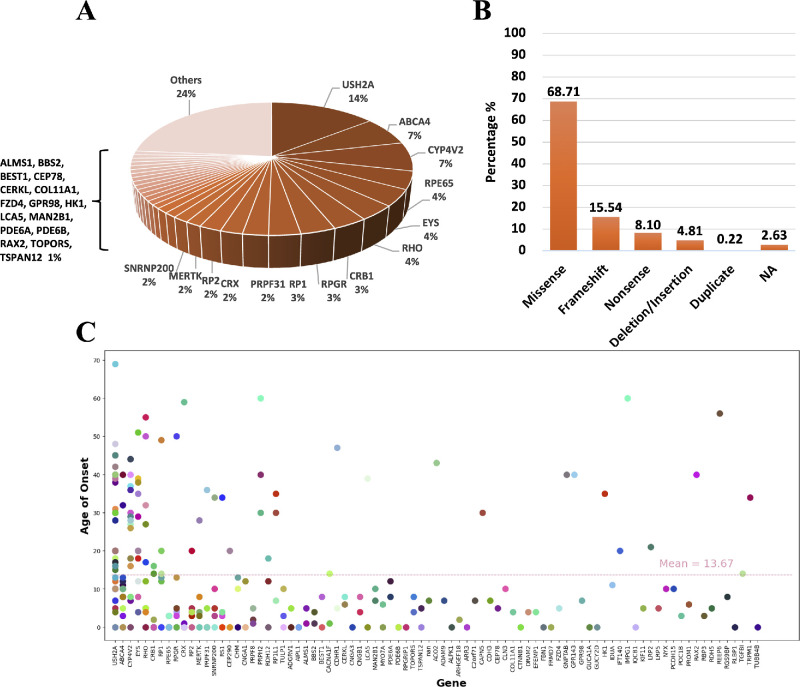
**Genetic characteristics of all included patients.** (**A**) Distribution of the genotypes among 308 included patients. Pathogenic variants were identified across 91 genes, with mutations in the top 14 genes accounting for 59% of the cases. Others including *AIPL1*, *ALMS1*, *BBS2*, *BEST1*, *CEP78*, *CERKL*, *COL11A1*, *FZD4*, *GPR98*, *HK1*, *LCA5*, *MAN2B1*, *PDE6A*, *PDE6B*, *RAX2*, *TOPORS*, *TSPAN12*, *ACO2*, *ADAM9*, *ALPK1*, *ARHGEF18*, *ARR3*, *C2orf71*, *CAPN5*, *CDH3*, *CLN3*, *CTNNB1*, *DRAM2*, *EFEMP1*, *FBN1*, *FRMD7*, *GNPTAB*, *GPR143*, *GUCA1A*, *GUCY2D*, *IDUA*, *IMPG1*, *IMPG2*, *IQCB1*, *KIF11*, *KLHL7*, *LRMDA*, *LRP2*, *LRP5*, *NYX*, *PAX6*, *PCDH15*, *PEX1*, *POC1B*, *PROM1*, *RBP3*, *RDH5*, *REEP6*, *RGS9BP*, *RLBP1*, *SMARCA4*, *TGFBI*, *TRPM1*, *UBB4B*, and *VAX2*. (**B**) Characteristics of the structural classifications of mutated proteins. (**C**) Correlation between onset age and genotype distribution across patients.

A total of 601 eyes from 308 consecutive patients with IRD were finally enrolled in this study, including 179 men and 129 women, with an average age at diagnosis of 38.45 ± 17.97 years and a mean age of onset of 13.67 ± 15.53 years. Table 1 presents the baseline demographics and clinical characteristics of the cohort, whereas Figure 2 provides an overview of the genetic findings. These eyes were then divided into 2 categories on the basis of OCT and FAF results: 195 eyes (105 patients) were included in the foveal-sparing group and 406 eyes (203 patients) were included in the foveal-involved group. The nine most frequently identified genes were *USH2A* (14.42% of all patients), *ABCA4* (7.01% of all patients), *CYP4V2* (7.01% of all patients), *RPE65* (4.29% of all patients), *EYS* (3.99% of all patients), *RHO* (3.68% of all patients), *CRB1* (3.37% of all patients), *RPGR* (3.37% of all patients), and *RP1* (3.07% of all patients).

**Table 1.** Comparison Between Foveal-Sparing Eyes and Foveal-Involved Eyes

Parameter	Overall	FS	Non-FS	*P* Value
No. patients, %	308	105	203	/
No. eyes, %	601	195	406	/
Gender, M/F^†^	179/129	58/47	121/82	0.54
Age, mean ± SD, y^*^	38.45 ± 17.97	36.61 ± 17.55	39.40 ± 18.16	0.20
Onset age, mean ± SD, y^*^	13.67 ± 15.53	18.69 ± 17.24	11.11 ± 13.95	**<0.01**
Grouping by known disease duration^†^				
<10 y, %	55 (17.9)	30 (28.6)	25 (12.3)	**<0.01**
10–30 y, %	122 (39.6)	46 (43.8)	76 (37.4)	
>30 y, %	90 (29.2)	19 (18.1)	71 (35.0)	
NA	41 (13.3)	10 (9.5)	31 (15.3)	
BCVA logMAR^*^	1.19 ± 0.92	0.40 ± 0.41	1.58 ± 0.85	**<0.01**
Choroidal atrophy, %^§^	48 (8.0)	4 (2.1)	44 (10.8)	**<0.01**
CFT, µm^*^	213.70 ± 87.39	237.15 ± 82.06	198.80 ± 87.53	**<0.01**
Atrophy size, mm^2^^*^	40.50 ± 32.52	33.75 ± 28.51	44.86 ± 34.26	**0.01**
AF pattern^†^				
Pattern 1	57 (9.5)	39 (20.0)	18 (4.4)	**<0.01**
Pattern 2	124 (20.6)	76 (39.0)	48 (11.8)	
Pattern 3	217 (36.1)	29 (14.9)	188 (46.3)	
Diagnosis^§^				
RP	156 (50.6)	66 (62.9)	90 (44.3)	**<0.01**
STGD/CORD	32 (10.4)	3 (2.9)	29 (14.3)	**<0.01**
BCD	19 (6.2)	5 (4.8)	14 (6.9)	0.46
LCA	25 (8.1)	4 (3.8)	21 (10.3)	**0.05**
Others	76 (24.7)	27 (25.7)	49 (24.1)	0.76

BCD, Bietti crystalline dystrophy; BCVA, best-corrected visual acuity; CFT, central foveal thickness; CORD, cone rod dystrophy; FS, foveal sparing; IOP, intraocular pressure; LCA, Leber congenital amaurosis; non-FS, foveal involved; RP, retinitis pigmentosa; STGD, Stargardts’ disease.

The *P* values in bold represent statistical significance.

* Students *t*-tests.

† Chi-squared test.

§ Fisher’s exact test.

### Characteristics of Foveal Sparing Eyes

In our cohort, a total of 15 patients exhibited unilateral foveal sparing, whereas 90 patients showed bilateral foveal sparing. The genotype distribution within the unilateral foveal sparing subgroup was as follows: *RPGR* in 3 of 15 cases (20%), *CYP4V2* in 2 of 15 cases (13.3%), and 1 case each associated with *RLBP1*, *MERTK*, *COL11A1*, *CRX*, *USH2A*, *SNRNP200*, *CACNA1F*, *SMARCA4*, *CNGA3*, and *CRB1*.

Compared with foveal-involved eyes, foveal-sparing eyes presented with a later onset age (18.69 ± 17.24 years vs. 11.11 ± 13.95 years, *P* < 0.01), smaller proportion of cases with longer disease duration distribution (*P* < 0.01), better BCVA (0.40 ± 0.41 logMAR vs. 1.58 ± 0.85 logMAR, *P* < 0.01), thicker central foveal thickness (CFT; 237.15 ± 82.06 µm vs. 198.80 ± 87.53 µm, *P* < 0.01), lower frequency of choroidal atrophy (*P* < 0.01), and smaller atrophy size (33.75 ± 28.51 mm^2^ vs. 44.86 ± 34.26 mm^2^, *P* = 0.01). There was no significant difference in gender distribution and current age between the two groups. We further analyzed morphological characteristics of retinal microstructures in foveal sparing group by SD-OCT in detail, including ELM, EZ, and IZ. Structure of EZ (96.4%) was preserved in most cases, followed by the ELM structure (82.6%), whereas IZ structure was preserved in the fewest cases (30.3%). Average maximum preserved length of all foveal sparing eyes on the OCT of ELM, EZ, and IZ were 2.81 ± 2.35 mm, 2.73 ± 2.52 mm, and 1.01 ± 2.23 mm, respectively. Ophthalmic details are listed in Table 2.

**Table 2.** Demographic and Clinical Characteristics of RP Patients With Foveal Sparing

				*P* Value		
Parameter	RP	*USH2A*-RP	*EYS*-RP	RP vs. *USH2A*	RP vs. *EYS*	*USH2A* vs. *EYS*
No. patients, %	73	22	9			
No. eyes, %	137	43	18			
Gender, M/F^§^	38/35	9/13	5/4	0.36	0.84	0.46
Age, mean ± SD, y^*^	39.82 ± 15.17	42.18 ± 14.99	41.56 ± 13.61	0.52	0.74	0.91
Onset age, mean ± SD, y^*^	20.25 ± 17.06	25.10 ± 16.32	26.63 ± 16.58	0.25	0.32	0.82
BCVA, logMAR^*^	0.32 ± 0.30	0.32 ± 0.26	0.24 ± 0.33	0.89	0.34	0.31
Choroidal atrophy, n (%)^§^	4 (0.7)	2 (4.7)	1 (5.6)	0.54	0.51	0.88
Structure residual at the fovea^†^^,^^§^						
ELM	118 (86.13)	37 (86.0)	15 (83.3)	0.99	0.75	0.79
EZ	133 (97.10)	41 (95.3)	17 (94.4)	0.58	0.55	0.88
IZ	41 (29.92)	11 (25.6)	1 (5.6)	0.58	0.03	0.07
Maximum residual layers of EZ^*^	7.13 ± 5.37	5.98 ± 4.14	7.65 ± 4.90	0.20	0.71	0.19
Maximum residual length, mm^*^						
ELM	2.75 ± 2.05	2.39 ± 1.56	2.90 ± 1.92	0.43	0.79	0.39
EZ	2.32 ± 1.97	1.88 ± 1.20	2.32 ± 1.86	0.17	0.99	0.29
IZ	0.78 ± 1.87	0.41 ± 0.73	0.08 ± 0.29	0.21	0.17	0.11
Residual EZ area, mm^2^^*^	7.47 ± 2.53	5.38 ± 6.27	8.23 ± 12.00	0.13	0.76	0.24
CFT, µm^*^	232.70 ± 57.74	217.67 ± 37.84	232.59 ± 59.95	0.11	0.99	0.25

ELM, external limiting membrane; EZ, ellipsoid zone; *EYS*-RP, *EYS* related retinitis pigmentosa; IZ, interdigitation zone; *USH2A*-RP, *USH2A* related retinitis pigmentosa.

* Students *t*-tests.

† Chi-squared test.

§ Fisher's exact test.

**Figure 3.**
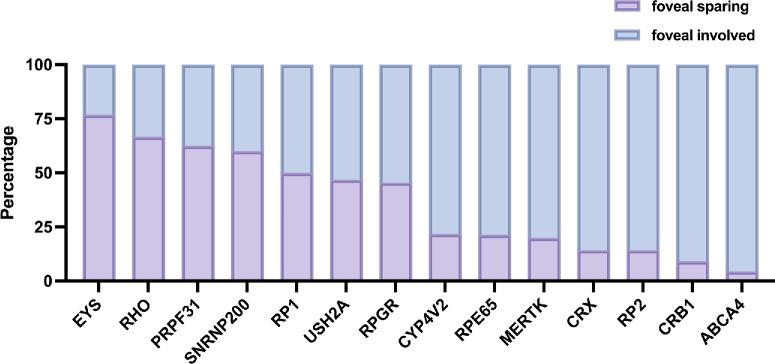
**Frequency of foveal sparing across causative genes in the analyzed cohort.** This histogram showed the distribution of foveal sparing in IRDs associated with the most prevalent genotypes identified in our cohort (*N* > 4). Bars in *purple* highlighted the proportion of patients with foveal sparing, whereas bars in *blue* indicate the proportion of patients with foveal involvement.

We further analyzed gene variants that appeared in more than five patients. Among all patients showing foveal sparing, the highest mutation frequency was *USH2A* (19.64%), followed by *EYS* (8.92%), *RHO* (7.14%), *CYP4V2* (4.46%), and *PRPF31* (4.46%), as shown in Supplementary Figure S2. The highest proportion of foveal sparing among included causative genetic variants was 76.92% in patients with *EYS* variants, followed by *RHO* (66.67%), *USH2A* (46.81%), PRPF31 (62.5%), *RP1* (55.56%), *RPGR* (45.45%) and *CYP4V2* (21.74%; the top 14 genes are listed in Fig. 3).

**Figure 4.**
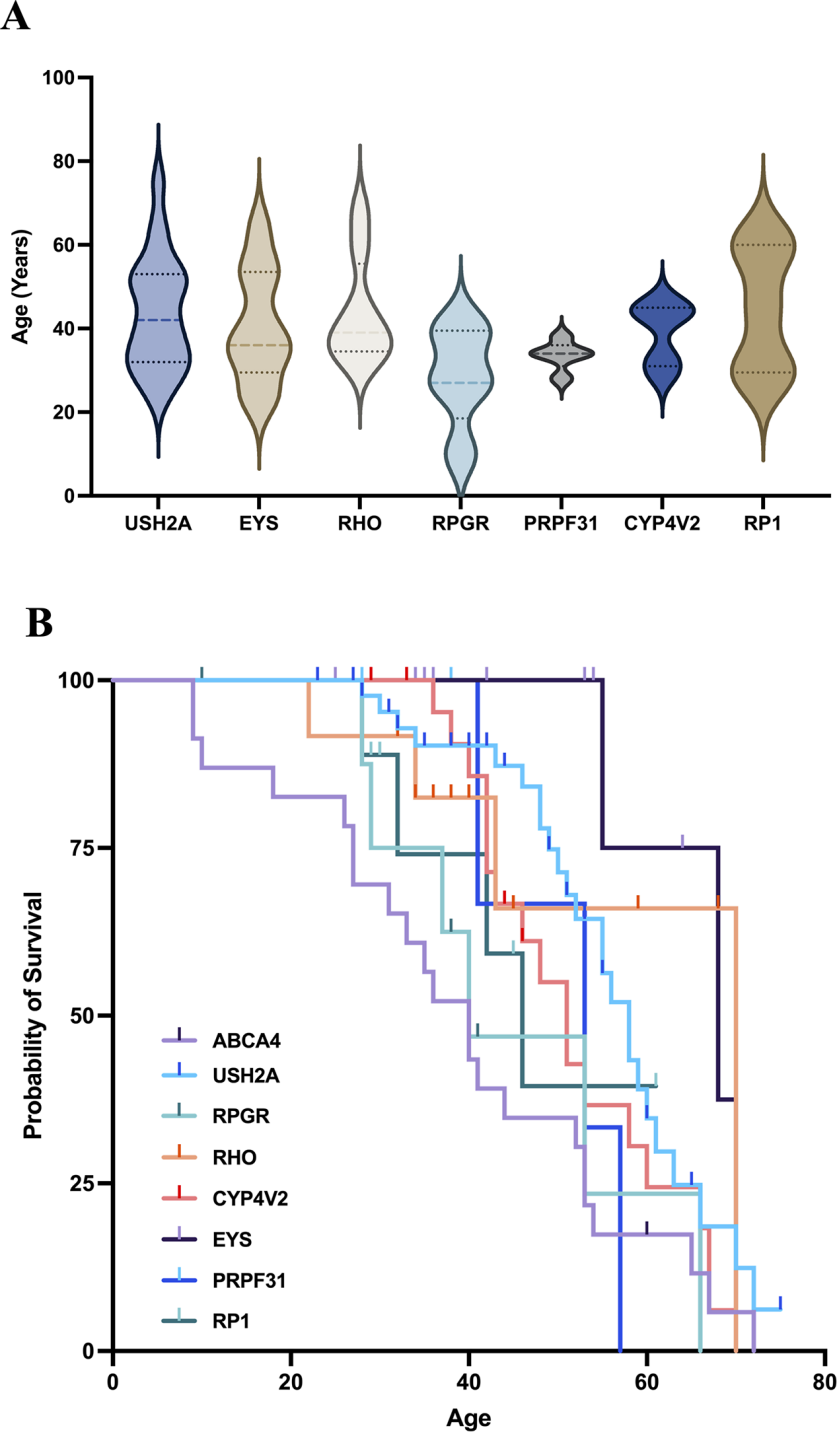
**Age distribution of foveal sparing and progression to foveal involvement across genotypes.** (**A**) Age distribution of patients with foveal sparing across causative genes represented by more than five individuals. (**B**) Survival curves corresponding to genotypes appearing in more than eight individuals in the overall cohort. Foveal involvement was the cutoff event.

To explore the differences in the natural course of foveal sparing among different genotypes, we finally included genotypes with more than five individuals showing foveal sparing and analyzed their age distributions (Fig. 4A). Among patients with foveal sparing, those with *RHO* and *RP1* mutations were the oldest, with mean ages of 44.00 ± 12.88 years and 44.8 ± 15.27 years, respectively. The mean age of patients with *USH2A* mutations was 43.67 ± 15.60 years, whereas that of patients with *EYS* mutations was 40.67 ± 13.84 years. Patients with *RPGR* mutations constituted the youngest group, with a mean age of 28.60 ± 12.18 years. We then performed survival analysis relative to age for each genotype to better illustrate the natural process of foveal preservation (Fig. 4B).

In our study, all patients with *USH2A* and *EYS* mutations who exhibited foveal sparing were clinically diagnosed with RP. To minimize phenotypic confounding, we limited our genotype-phenotype correlation to patients with an RP phenotype only. Within this RP cohort, we specifically compared patients showing foveal sparing with *USH2A* or *EYS* mutations, focusing on structural parameters such as residual EZ/IZ layers and central foveal thickness in order to determine the potential role of genotype. Our cohort showed no statistically significant differences between the *USH2A* and *EYS* groups in terms of age, BCVA, maximum residual layers of the EZ, maximum EZ length, or residual EZ area. The mean age was comparable between the two groups (*USH2A* = 42.18 ± 14.99 years; *EYS* = 41.56 ± 13.61 years, *P* > 0.05), as was BCVA (*USH2A* = 0.32 ± 0.26 logMAR; *EYS* = 0.24 ± 0.33 logMAR, *P* > 0.05). The maximum residual EZ layer count and EZ length did not differ significantly between the groups. Similarly, the residual EZ area was slightly higher in the *EYS* group (8.23 ± 12.00 mm²) than in the *USH2A* group (5.38 ± 6.27 mm²), but this difference was not statistically significant.

### Potential Prognostic Predictor for IRD

Given the variability in clinical features among eyes with IRD, we analyzed clinical information and retinal imaging features at initial diagnosis that have been previously reported to show good correlations with BCVA to assess their relationship (see Supplementary Table S1). Our analysis revealed that CFT (ρ = −0.185, *P* = 0.01), maximum residual layers of EZ (ρ = −0.244, *P* < 0.01), maximum residual ELM length (ρ = −0.337, *P* < 0.01), maximum residual EZ length (ρ = −0.239, *P* < 0.01), and residual EZ area (ρ = −0.261, *P* < 0.01) were negatively correlated with BCVA. However, the onset age, maximum residual IZ length, and atrophy area showed no significant correlations with BCVA.

**Figure 5.**
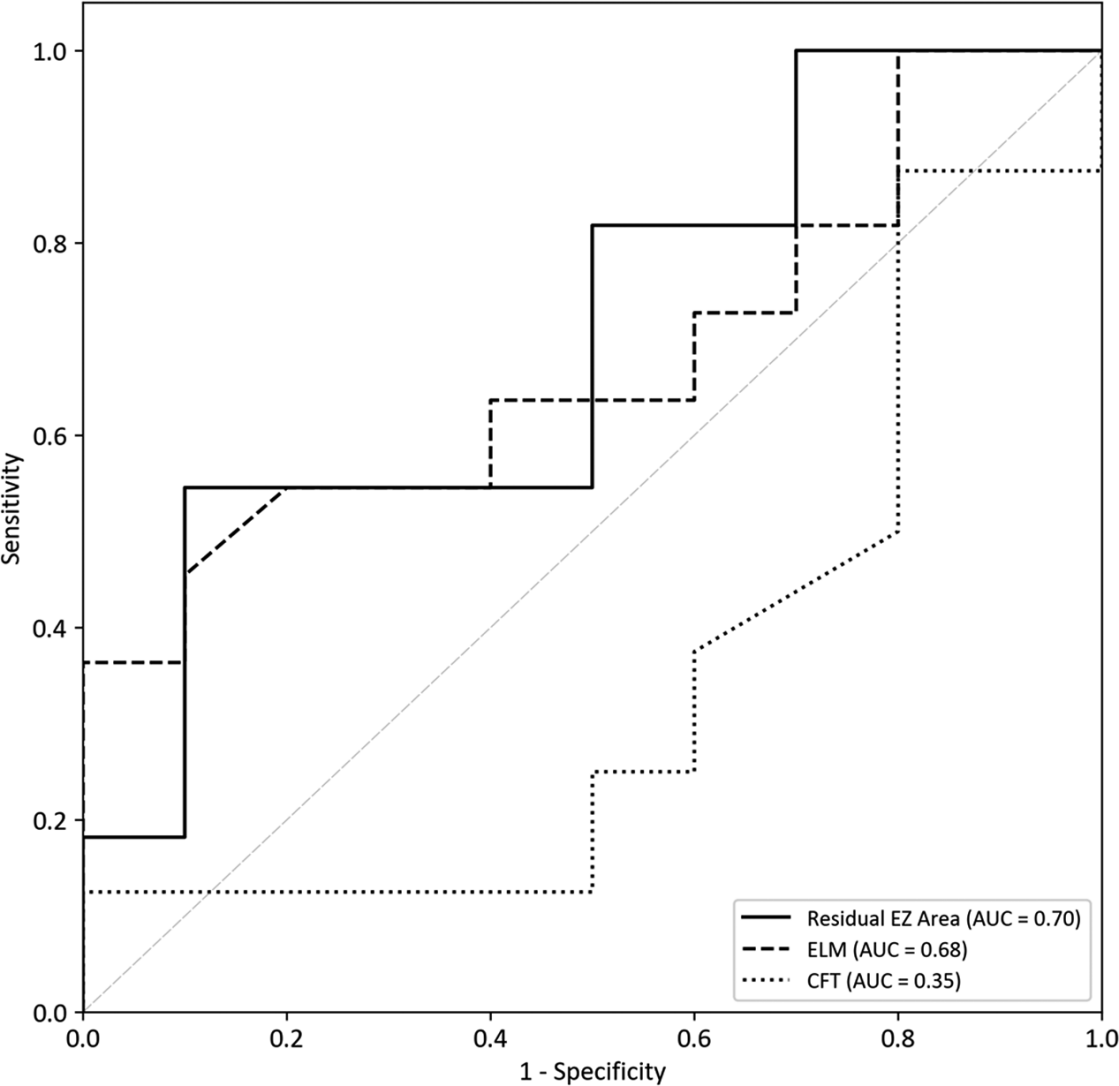
**Receiver operating characteristic (ROC) curves.** ROC curves were generated to evaluate whether baseline optical coherence tomography (OCT) parameters could predict the degree of visual acuity decline within 2 years. Among the parameters analyzed, the residual ellipsoid zone (EZ) area demonstrated the highest predictive performance.

To better predict BCVA deterioration in patients with IRD, we selected CFT, residual EZ area, and residual ELM, which showed the strongest correlations with BCVA in the Spearman correlation analysis, for subsequent ROC curve analyses. This analysis included 21 foveal sparing eyes from 12 patients with complete follow-up data over a period of 2 years. We then performed area under the curve (AUC) analysis to evaluate their predictive value (Fig. 5). BCVA reduction exceeding 0.1 logMAR was considered clinically significant. Notably, the residual EZ area exhibited an AUC of 0.70 (95% confidence interval [CI] = 0.417–0.933), indicating that its ability to predict visual deterioration was higher than that of CFT and residual ELM length. At an optimal cutoff point of 6.50, the sensitivity and specificity of the model was 50% and 90%, respectively, demonstrating a reliable ability to predict visual acuity changes based on residual EZ area.

## Discussion

Foveal sparing is characterized by retinal atrophy surrounding a relatively preserved fovea, thereby maintaining central visual acuity. In this retrospective cohort study, we investigated the mutational spectrum and clinical manifestations in 601 eyes with disease-causing monogenetic mutations, including 195 eyes exhibiting foveal sparing. Our work presented that: (1) patients with IRD with *USH2A* and *EYS* variants are more likely to develop foveal sparing and show a longer duration foveal sparing than those carrying other causative genes; and (2) among foveal sparing eyes, the residual EZ area may be a novel imaging marker for predicting BCVA deterioration throughout the progression of IRDs.

In our cohort, patients with *USH2A* and *EYS* mutations were more likely to present with foveal sparing, and their disease progression appeared to be relatively slower. The prevalence of *USH2A* and *EYS* mutations was the highest in our study, exceeding that of previously reported.^32^ Among all eyes with foveal sparing, *USH2A* and *EYS* mutations accounted for 19.64% and 8.92%, respectively. Foveal sparing eyes carrying *USH2A* and *EYS* mutations in our study were older than 40 years old, which was slightly older than the findings from a recent Asian population study.^33^ Additional comparison between *USH2A*- and *EYS*-related foveal sparing revealed no significant differences in structural preservation or clinical features. This indicated that clinical patterns may be consistent among genotypes with higher prevalence of preserved fovea. Notably, among patients with ABCA4 mutations, only 1 of 23 exhibited foveal sparing, a frequency lower than the prevalence of approximately 30% reported in previous studies in Caucasian patients.^19^^,^^21^ We speculate that this discrepancy may be due to the fact that our cohort was consisted of consecutive patients recruited within a defined time period, thereby reflecting only the distribution of *ABCA4* cases who visited our clinic during this interval, but not fully representing the overall prevalence of foveal sparing among Chinese patients with *ABCA4* mutations.

Although many researchers have observed the association between better central vision and preserved EZ length among patients with GA and IRD, no study has specifically established a residual EZ area as an OCT biomarker for this correlation in patients with IRD vision change.^16^^,^^21^^,^^31^ In our Spearman’s rank correlation analysis, we found that the preservation of CFT, maximum residual layers of EZ, maximum residual EZ length, and the residual EZ area were negatively related with BCVA, but positively related with visual acuity. In contrast, the retinal pigment epithelium loss observed on FAF did not serve as a significant predictor (*P* = 0.88). As previously reported, foveal sparing eyes in patients with IRD may develop photoreceptor-related symptoms, such as vision loss, visual field narrowing, and night blindness at a much later stage; whereas central vision as well as foveal and retinal structure can be well-preserved in the fourth and fifth decades of life.^34^ Consequently, SD-OCT-based measurement provides more detailed assessment for photoreceptors in foveal sparing eyes that cannot be achieved by FAF.

Our study then proposed an OCT-based imaging biomarker for predicting visual progression in patients with IRD. ROC curve analysis demonstrated that the residual EZ area has a moderate ability to predict visual acuity decline within 2 years, with an AUC of 0.70. A cutoff value of 6.50 mm² was identified as the optimal threshold for distinguishing patients whose BCVA decline remained within 0.1 logMAR from those who showed greater deterioration. The ROC curve analysis in this study provided a clinically meaningful measurement indicator that may help clinicians better understanding the natural course of IRD. Specifically, the cutoff value derived from the analysis can provide practical guidance for clinical decision making. Patients with residual EZ areas below the cutoff are at higher risk of visual decline and may require more frequent follow-up visits or earlier therapeutic interventions, whereas those above the cutoff can be monitored at longer intervals. Furthermore, this threshold is also of importance for determining treatment timing: in the context of future gene therapy or pharmacological trials, the cutoff may serve as an objective reference to identify patients who should receive earlier interventions. Although the predictive strength of the residual EZ area is not highly robust, it may still serve as a useful parameter for assessing disease progression and guiding clinical prognosis.

The close association between the residual EZ area and BCVA may be explained by differential vulnerability of photoreceptors and the anatomic distribution of retinal blood supply. S-cones and rods, absent at the foveal center, are considered to be more vulnerable to mitochondria-related damage, compared with M- and L-cones.^35^^–^^37^ Another hypothesis is that blood supply may contribute to foveal sparing. Our results showed that among all foveal sparing patients, those with *USH2A* alleles had a lower prevalence of choroidal atrophy (*P* < 0.01), indicating that choroidal blood flow may be involved in preserving retinal function in patients with *USH2A* mutations. Previous studies utilizing OCT angiography have reported a reduction in vascular density of deep capillary plexus and superficial capillary plexus of the macular vascular network among patients with IRD.^38^^–^^40^ Choriocapillaris in patients with *USH2A* variants has been shown to remain relatively preserved until late stages of the disease.^41^ We hypothesized that the development of foveal sparing may be influenced by varying sensitivities to ischemia and hypoxia of different types of photoreceptors, leading to differences in mitochondrial integrity. Thus, preservation of the EZ band is not only a morphologic basis for preserved visual function in patients with IRD, but also provides insights for exploring key pathological mechanisms of foveal sparing.

Although foveal sparing in RHO-related retinopathy is well understood, the underlying reason why *USH2A*- and *EYS*-related retinopathy are more likely to develop foveal sparing remains unknown. Previous studies have indicated that several IRDs are linked to demonstrate foveal sparing characteristics.^14^^,^^42^^,^^43^ In the meanwhile, we found that both the *USH2A* gene and *EYS* gene also locate at the connecting cilium (CC) in photoreceptors and play an important role in the development and structural maintenance and in these cells.^44^^,^^45^ Mutations of genes involved in the development of ciliary structure are known to cause Bardet‐Biedl syndrome (BBS). Several cases of BBS have been reported to present with the foveal sparing phenotype, suggesting another underlying explanation. Researchers have revealed that nonsense-mediated decay may play a significant role in phenotypic differences in *EYS*-related retinal dystrophies because the truncated EYS protein can still retain some functionality, maintaining the health of photoreceptors.^46^^,^^47^ Therefore, the likely cause of foveal sparing in eyes with *EYS* mutations may be attributed to mild protein dysfunction rather than the toxicity of unfolded proteins.

Our study also identified the residual EZ area as a novel imaging predictor of visual deterioration in IRD natural process. Although recent researches have considered EZ/ELM disruption or retina layer thickness as robust SD-OCT–based imaging markers for predicting visual outcome and lesion progress in eyes,^48^ these markers only supply linear and localized assessments of the retina, which may not adequately capture the full spectrum of microstructural changes. Our finding provides a volumetric approach based on SD-OCT measurements, allowing a more comprehensive depiction of photoreceptor loss and central vision prognosis. Clinically, this marker serves as a practical tool for disease diagnosis and identifying therapeutic windows.

The limitations of our study include the absence of a large-scale longitudinal cohort and the lack of electroretinography data for a more comprehensive assessment of photoreceptor function. Because only consecutive patients within a specific time window were included, the findings merely reflect the clinical characteristics of patients presenting to our center during that period and may not be sufficiently large to permit subgroup analyses about progression and whether fast or slow progressors can be identified. To better evaluate the characteristics of foveal sparing among diverse causative genes, longitudinal studies with larger sample sizes are required to further validate the predictive value and improve the reliability of these findings.

## Conclusions

Our study highlights that the foveal sparing phenomenon, especially preservation of the EZ band, is closely related to the long-term visual prognosis of patients with IRD. *EYS* and *USH2A* are closely associated with the foveal sparing phenomenon. Notably, we proposed a new SD-OCT–based predictive indicator that can predict the long-term visual outcome of patients with IRD. These findings provide a valuable tool for understanding the progression of IRD and capturing the treatment window, which may shed light to the etiology of retina degeneration in patients with IRD. Further cohort validation is essential to confirm whether the proposed indicator can truly serve as a reliable predictor.

## Supplementary Material

Supplement 1
