# Supplementary material for: Clinical and Molecular Characteristics of Foveal Sparing Phenotype in Chinese Patients With Inherited Retinal Diseases
Source: Transl Vis Sci Technol. 2026 Feb 17;15(2):18. doi: 10.1167/tvst.15.2.18 (PMC12922713; doi:10.1167/tvst.15.2.18)
Supplement: Supplement 1 [file tvst-15-2-18_s001.pdf]

Supplementary material

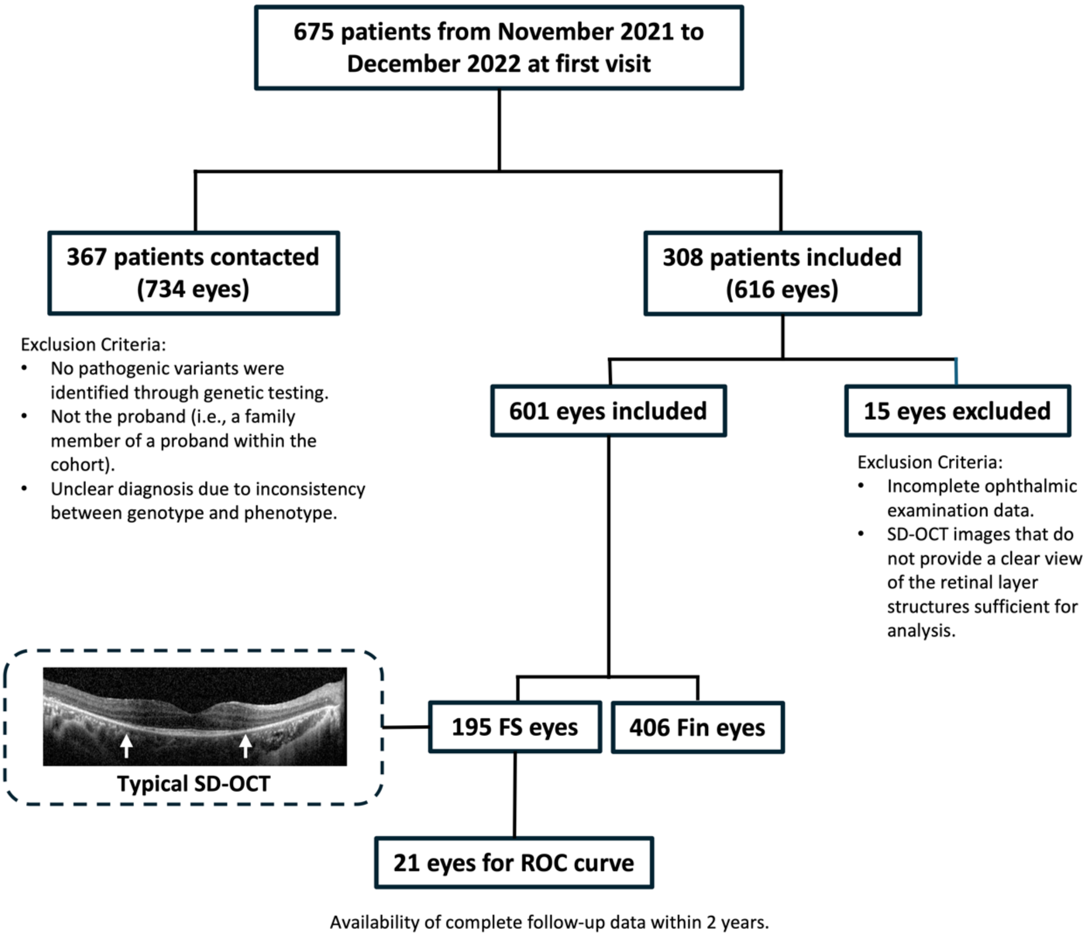

Figure S1. Flow chart of patient inclusion. All inclusion and exclusion criteria are outlined within the diagram. The white arrow in the representative SD-OCT image marks the end of the residual EZ band.

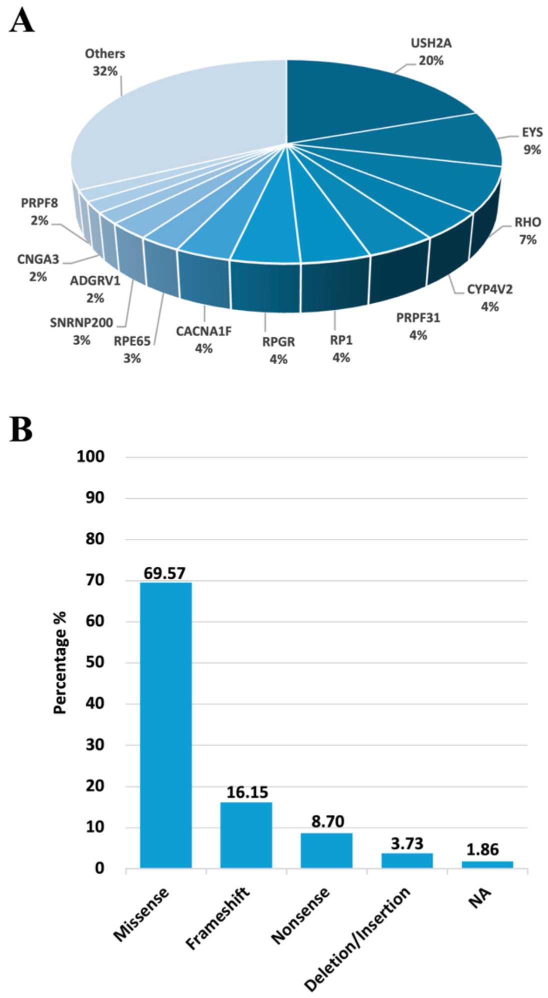

Figure S2. Genetic characteristics of foveal sparing patients.

(A) Distribution of the 105 included patients with foveal sparing eyes. Pathogenic variants were identified across 49 genes, with mutations in the top 13 genes accounting for 68% of the cases. Others including ABCA4, ALPK1, CAPN5, CDHR1, CEP290, CEP78, CHM, CNGA1, CNGB1, COL11A1, CRB1, CRX, FZD4, GNPTAB, GPR143, GPR98, IFT140, LRP5, MERTK, MYO7A, NYX, PDE6A, POC1B, PRPH2, RAX2, RDH5, REEP6, RGS9BP, RLBP1, RP1L1, RP2, RS1, SMARCA4, TOPORS, TRPM1, TULP1. (B) Characteristics of the structural classifications of mutated proteins

Table S1. Correlation Analysis between Clinical Features at initial diagnosis and BCVA (LogMAR)

**Variable 1**	**Variable 2**	**Spearman Correlation Coefficient ρ**	**P Value**
BCVA (LogMAR)	Onset Age	-0.201	0.08
	CFT	-0.185	**0.01**
	Maximum residual layers of EZ	-0.244	**<0.01**
	Maximum residual ELM length	-0.337	**<0.01**
	Maximum residual EZ length	-0.239	**<0.01**
	Maximum residual IZ length	-0.147	0.06
	Residual EZ Area	-0.261	**<0.01**
	Atrophy Area	0.017	0.88

Abbreviations: BCVA=Best-Corrected Visual Acuity; ELM=External Limiting Membrane; EZ=ellipsoid zone; IZ=Interdigitation zone; CFT=Central Foveal Thickness.
